# The Association Study of *IL-23R* Polymorphisms With Cerebral Palsy in Chinese Population

**DOI:** 10.3389/fnins.2020.590098

**Published:** 2020-11-25

**Authors:** Yangong Wang, Yiran Xu, Yangyi Fan, Dan Bi, Juan Song, Lei Xia, Qing Shang, Chao Gao, Xiaoli Zhang, Dengna Zhu, Yimeng Qiao, Yu Su, Xiaoyang Wang, Changlian Zhu, Qinghe Xing

**Affiliations:** ^1^Institutes of Biomedical Science and Children's Hospital, Fudan University, Shanghai, China; ^2^Henan Key Laboratory of Child Brain Injury, Department of Pediatrics, The 3rd Affiliated Hospital of Zhengzhou University and Institute of Neuroscience, Zhengzhou, China; ^3^Department of Pediatrics, Qilu Hospital of Shandong University, Jinan, China; ^4^Department of Pediatrics, Children's Hospital of Zhengzhou University and Henan Children's Hospital, Zhengzhou, China; ^5^Child Rehabilitation Center, The 3rd Affiliated Hospital of Zhengzhou University, Zhengzhou, China; ^6^Department of Women's and Children's Health, Karolinska Institutet, Stockholm, Sweden; ^7^Center for Brain Repair and Rehabilitation, Institute of Neuroscience and Physiology, Sahlgrenska Academy, University of Gothenburg, Gothenburg, Sweden; ^8^Shanghai Center for Women and Children's Health, Shanghai, China

**Keywords:** cerebral palsy, inflammatory cytokines, interleukin, gene polymorphism, IL23R

## Abstract

**Background:** Cerebral palsy (CP) is a syndrome of non-progressive motor dysfunction caused by early brain development injury. Recent evidence has shown that immunological abnormalities are associated with an increased risk of CP.

**Methods:** We recruited 782 children with CP as the case group and 770 healthy children as the control group. The association between *IL-23R* single nucleotide polymorphisms (SNPs; namely, rs10889657, rs6682925, rs1884444, rs17375018, rs1004819, rs11805303, and rs10889677) and CP was studied by using a case–control method and SHEsis online software. Subgroup analysis based on complications and clinical subtypes was also carried out.

**Results:** There were differences in the allele and genotype frequencies between CP cases and controls at the rs11805303 and rs10889677 SNPs (*P*allele = 0.014 and 0.048, respectively; *P*genotype = 0.023 and 0.008, respectively), and the difference in genotype frequency of rs10889677 remained significant after Bonferroni correction (*P*genotype = 0.048). Subgroup analysis revealed a more significant association of rs10889677 with CP accompanied by global developmental delay (*P*genotype = 0.024 after correction) and neonatal encephalopathy (*P*genotype = 0.024 after correction).

**Conclusion:** The present results showed a significant association between *IL-23R* and CP, suggesting that *IL-23R* may play a potential role in CP pathogenesis.

## Introduction

Cerebral palsy (CP) is a central motor disorder syndrome that manifests with abnormal muscle tension and motor function (Cheng et al., 2011; Wu et al., 2011). Individuals with CP often exhibit sensory, perceptual, cognitive, communication, and behavioral disorders, as well as epilepsy and secondary musculoskeletal problems (Beliakoff and Sun, 2006). Although perinatal medicine has developed rapidly in recent years, epidemiological studies have shown that the incidence of CP has remained stable at 2–3.5 children out of every 1,000 children (Pennington et al., 2013). CP has been a prominent disease among children's disabilities for a long time due to a lack of effective treatments (Tator et al., 2007; Tatla et al., 2013). CP seriously affects the quality of life of individuals and also brings a heavy financial burden to families and society (Duval et al., 2003; Yunus and Lima, 2009). The identification of its etiology and pathogenesis is essential to the prevention and control of CP.

CP is caused by non-progressive brain damage during the development of the fetus or infant, which can be divided into congenital and acquired damage. Congenital non-progressive injuries are caused by prenatal developmental defects, such as genetic defects, developmental defects, malformations, intrauterine infections, etc. Acquired injuries are caused by postpartum acquired factors, such as premature labor, asphyxia, hypoxic–ischemic encephalopathy (HIE), low birth weight (LOW), and pathogenic jaundice (Sachdev et al., 2001; Jacobsson and Hagberg, 2004). Increasing evidence now indicates that genetic factors are likely to play an important role in CP pathogenesis. In general, CP is regarded as the result of the combined effects of multiple genes and environmental factors. Moreover, CP has been reported to have multiple susceptibility genes, including *IL-6, NOS1, OLIG2, ATG5*, and *ATG7* (Xu et al., 2017; Yu et al., 2018; Sun et al., 2019; Xia et al., 2019).

A great deal of evidence suggests that neuroinflammation has been found to participate in, modulate, and even induce the pathological process of immature brain injury and various cytokines have been associated with CP and neurodevelopmental disability. Previous studies have found that immature brain injury induced by secondary inflammation is one of the important pathological mechanisms of hypoxic–ischemic brain injury (Elovitz et al., 2011; Albertsson et al., 2014; Marshall and Plotkin, 2019). Abnormal activation of cytokines can cause brain damage, and fetuses are more likely to produce inflammatory reactions or brain damage after being affected by inflammatory cytokines due to immature brain development, thus further affecting the normal development of the brain. At present, many inflammatory cytokines have been reported to be significantly associated with CP or neurodevelopmental disorders, such as *IL-6, IL-8, IL-10*, and *IL-17* (Strle et al., 2001; Chiricozzi et al., 2011; Chen et al., 2013; Magalhaes et al., 2019).

Interleukin-23 (*IL-23*), also known as p19, is a member of the *IL-12* heterodimer cytokine family (Oppmann et al., 2000), which is mainly produced by activated dendritic cells, macrophages, and monocytes. *IL-23* plays an important role in the regulation of tissue homeostasis and congenital or adaptive immunity. IL-23 is involved in the pathogenesis of many chronic inflammatory diseases, such as psoriasis, arthritis, inflammatory bowel disease, and multiple sclerosis (MS). Drugs targeting IL-23 have been used in clinical research regarding immunologic diseases (Wiekowski et al., 2001; Schon and Erpenbeck, 2018). IL-23 binds to the IL-23 receptor (*IL-23R*) through its N-terminal immunoglobulin domain, which activates downstream signaling pathways and exerts biological functions. Human IL-23 receptors (*IL-23R*) are mainly expressed in activated memory T cells, natural killer (NK) cells, and intrinsic immune cells (ILCs). Its extracellular domain contains a signal sequence, one N-terminal Ig-like domain, and two cytokine receptor domains. In a genome-wide association study in 2006, *IL-23R* was significantly associated with Crohn's disease, an inflammatory bowel disease. The A allele of rs11209026, a low-frequency *IL-23R* variant, was negatively correlated with Crohn's disease (Bloch et al., 2018). At the same time, some studies have shown that *IL-23R* mutation is significantly correlated with inflammatory demyelinating diseases, such as MS (Li et al., 2016).

Based on the above information, we speculate that *IL-23R* may be associated with susceptibility to CP, but no relevant studies have been reported thus far. Then, we used a case–control study to explore the possible association of *IL-23R* with CP, which will provide genetic evidence for evaluating the role of *IL-23R* in the etiology of CP and its related potential mechanisms.

## Materials and Methods

### Participants

In this study, 782 children with CP and 771 healthy controls were recruited from the centers for CP rehabilitation and Child Healthcare Departments in the Third Affiliated Hospital of Zhengzhou University, Zhengzhou Children's Hospital. This study was approved by the ethics committee of Zhengzhou University. The guardians of these participants provided written informed consent. The case group comprised 542 males (69.3%) and 240 females (30.7%), and the mean age was 18.5 ± 15.4 months. The control group comprised 771 healthy children, including 515 males (66.8%) and 256 females (33.2%), and the mean age was 19.3 ± 16.8 months (Table 1).

**Table 1:** Clinical characteristics of all participants.

**Characteristic**	**CP cases (*n* = 782)**	**Controls (*n* = 771)**
Sex (male:female)	542:240	515:256
Preterm (<37 weeks)	47	10
<2,500 g	40	2
Birth asphyxia	234	13
**Type of CP**		
Spastic CP	522	NA
CP with quadriplegia	284	NA
CP with diplegia	126	NA
**Complications**		
CP with PVL	67	NA
CP with HIE	108	NA
CP with GDD	299	NA
Type of CP	310	NA
**Maternal factors**		
PROM	71	26
TPL	58	0
PIH	26	7

*CP, cerebral palsy; PVL, periventricular leukomalacia; HIE, hypoxic–ischemic encephalopathy; GDD, global developmental delay; PROM, premature rupture of membrane; TPL, threatened premature labor; PIH, pregnancy-induced hypertension*.

### CP Diagnosis, Classification, and Exclusion Criteria

In the case group, we excluded children diagnosed with congenital metabolic diseases and myopathy as well as children with a family history of nervous system diseases. Pediatric rehabilitation specialists confirmed the CP diagnosis using standard criteria related to non-progressive disorders of movement control and posture (Rosenbaum et al., 2007). All participants received a detailed clinical evaluation with comprehensive pre-test counseling.

The available clinical information included demographic variables, such as sex, gestational age, mode of delivery, singletons, and twins, as well as the known risk factors [such as pregnancy-induced hypertension (PIH), perinatal asphyxia, and threatened premature labor], CP complications [such as global developmental delay (GDD)], and neonatal complications (such as HIE).

GDD diagnosis is limited to individuals under the age of 5 years old when the clinical severity level cannot be reliably assessed during early childhood. GDD is diagnosed when an individual fails to meet the expected developmental milestones in several areas of intellectual functioning and applies to individuals who are unable to undergo systematic assessments of intellectual functioning, including children who are too young to participate in standardized testing. Neonatal encephalopathy (NE) is a clinical syndrome that includes HIE, intracranial hemorrhage, various metabolic disorders, neurodegenerative diseases, and so on; its diagnosis requires at least two senior neonatologists.

### Genotyping and Statistical Analysis

**Figure 1:**
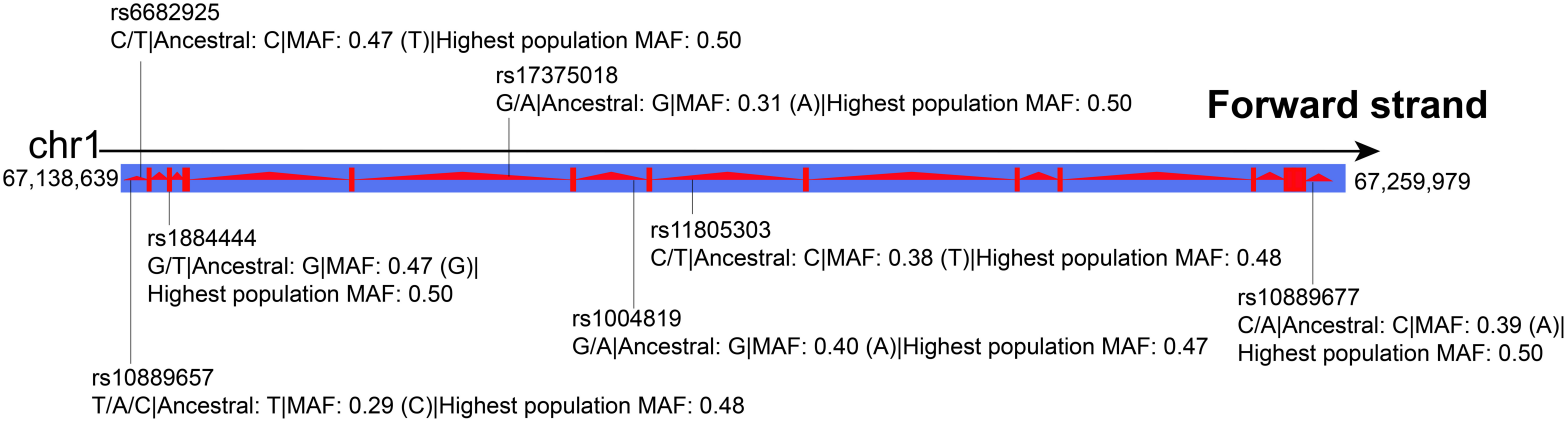
SNPs in *IL-23R*. Highest population minor allele frequency: highest minor allele frequency observed in any population including 1,000 Genomes Phase 3, ESP, and genomAD.

Peripheral blood samples were obtained from the subjects for genomic DNA extraction. According to the single nucleotide polymorphism (SNP) location in *IL-23R*, a minor allele frequency (MAF) >0.1, and potential function, we selected seven SNPs (rs10889657, rs6682925, rs1884444, rs17375018, rs1004819, rs11805303, and rs10889677, Figure 1) as candidates and genotyped them by the MassARRAY system. Shanghai Perchant Biotechnology Co., Ltd. synthesized primers and probes.

We performed statistical analysis with SHEsis, an online program (http://analysis.bio-x.cn/) that can test Hardy–Weinberg equilibrium (HWE) and linkage disequilibrium (LD) and calculate allele frequencies, genotype frequencies, and haplotype frequencies for each SNP locus in the case group and control group. The *P* values were two-tailed, and we considered *P* < 0.05 to be significant. We also calculated the odds ratio (OR) and its 95% confidence interval (CI). We employed the Bonferroni correction to account for multiple testing on each individual SNP and haplotype. We used the G^*^power 3.1 software to evaluate the statistical efficacy.

## Results

### Overall Analysis

**Figure 2:**
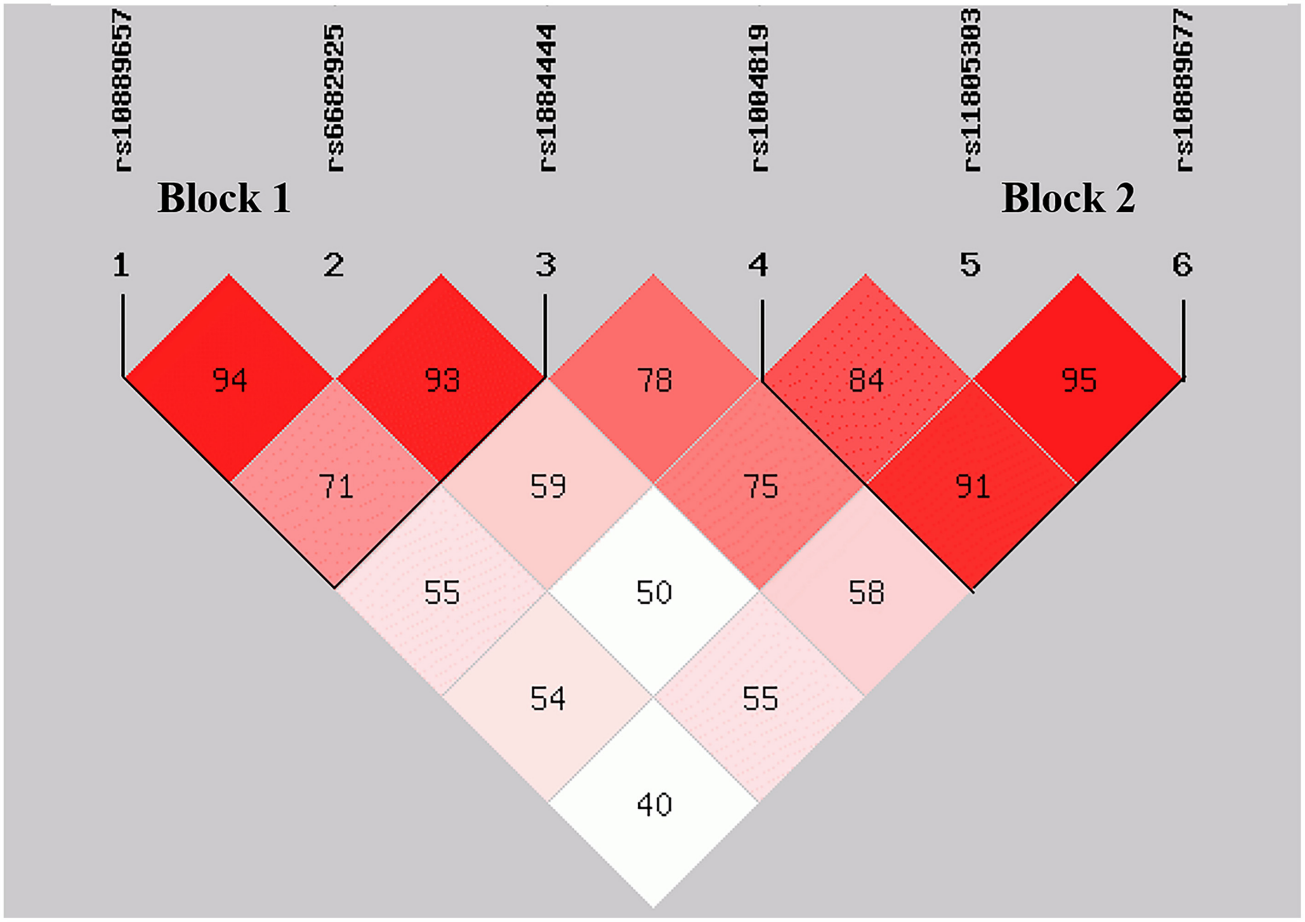
Distribution of blocks defined by linkage disequilibrium scores of six SNPs in *IL-23R*. The data indicate D′ values.

By performing power calculations, the sample size utilized in the present study has >85% power to detect a significant association (α < 0.05) when using an effect size index of 0.1. The genotype distributions of rs17375018 among control subjects showed a significant deviation from HWE (*P* = 0.011); therefore, we only analyzed the remaining six SNPs, namely, rs10889657, rs6682925, rs1884444, rs1004819, rs11805303, and rs10889677. There were two linked LD blocks with coefficient D′ value more than 0.8 (Figure 2). Block 1 included rs10889657, rs6682925, and rs1884444, whereas block 2 comprised rs1004819, rs11805303, and rs10889677.

For all subjects, the allele frequencies of rs11805303 (*P* = 0.014) and rs10889677 (*P* = 0.048) and the genotype frequencies of rs10889657 (*P* = 0.028), rs11805303 (*P* = 0.023), and rs10889677 (*P* = 0.008) were significantly different between CP patients and controls. After Bonferroni correction, only the rs10889677 AA genotype frequency was significantly more enriched in CP children than in controls (OR = 1.178, 95% CI = 1.002–1.386, *P*c = 0.048) (Table 2).

**Table 2:** Allele and genotype frequency analysis of SNPs of *IL-23R*.

**Group**	**Allele frequency**		***p* value**	**OR [95% CI]**	**Genotype frequency**			***p-*value**	**H-W**
rs10889657	C	T			C/C	C/T	T/T		
CP	347 (0.267)	953 (0.733)	0.09	0.861 [0.725–1.023]	59 (0.091)	229 (0.352)	362 (0.557)	0.028^a^	0.011
Control	372 (0.297)	880 (0.703)			53 (0.085)	266(0.425)	307 (0.490)		0.665
rs6682925	C	T			C/C	C/T	T/T		
CP	625 (0.422)	855 (0.578)	0.406	0.940 [0.813–1.088]	133 (0.180)	359 (0.485)	248 (0.335)	0.681	0.876
Control	634 (0.437)	827 (0.563)			139 (0.189)	365 (0.497)	231 (0.314)		0.807
rs1884444	G	T			G/G	G/T	T/T		
CP	517 (0.344)	985 (0.656)	0.169	0.900 [0.776–1.046]	91 (0.121)	335 (0.446)	325 (0.433)	0.275	0.744
Control	556 (0.368)	954 (0.632)			97 (0.128)	362 (0.479)	296 (0.392)		0.402
rs1004819	A	G			A/A	A/G	G/G		
CP	532 (0.566)	408 (0.434)	0.05	1.194 [1.000–1.425]	168 (0.357)	196 (0.417)	106 (0.226)	0.063	0.001
Control	544 (0.522)	498 (0.478)			150 (0.288)	244 (0.468)	127 (0.244)		0.16
rs11805303	C	T			C/C	C/T	T/T		
CP	628 (0.415)	884 (0.585)	0.014^b^	0.835 [0.723–0.964]	150 (0.198)	328 (0.434)	278 (0.368)	0.023^c^	0.003
Control	696 (0.460)	818 (0.540)			167 (0.221)	362 (0.478)	228 (0.301)		0.304
rs10889677	A	C			A/A	A/C	C/C		
CP	1121 (0.750)	373 (0.250)	0.048^d^	1.178 [1.002–1.386]	429 (0.574)	263 (0.352)	55 (0.074)	0.008^e^	0.099
Control	1079 (0.718)	423 (0.282)			378 (0.503)	323 (0.430)	50 (0.067)		0.085

OR, odds ratio; CI, confidence interval.The P values corrected by Bonferroni are

a *0.168*,

b *0.084*,

c *0.138*,

d *0.288*,

e *0.048*.

Haplotype analysis is a powerful strategy to determine whether or not the above-mentioned CP-associated SNPs have a greater effect when analyzed together. Hence, we performed a haplotype analysis of rs11805303 and rs10889677 SNPs. The haplotypes CC (*P* = 0.036) and TA (*P* = 0.013) presented a significant association with CP; the positive association of TA with CP was significant even after Bonferroni correction (*P* = 0.039). Furthermore, there was a statistically significant global effect of the haplotype (*P* = 0.039) (Table 3).

**Table 3:** Haplotype analysis of rs11805303 and rs10889677 SNPs.

**Haplotype**	**Case (frequency)**	**Control (frequency)**	***P* value**	**OR (95% CI)**
CA	250.91 (0.171)	271.19 (0.183)	0.413244	0.924 (0.765–1.117)
CC	356.09 (0.242)	410.81 (0.277)	0.036346	0.838 (0.711–0.989)
TA	850.09 (0.578)	793.81 (0.536)	0.013013	1.203 (1.040–1.392)
Fisher's *P* value			0.039	
Pearson's *P* value			0.039	

### Subgroup Analysis

CP is a highly heterogeneous condition that likely has multiple etiologies. It is reasonable to speculate that the broad clinical spectrum of CP can be at least partially attributed to considerable genetic heterogeneity. Therefore, we conducted subgroup analysis according to clinical phenotypes. There was a significant association of CP + GDD with rs10889657, rs1004819, rs11805303, and rs10889677, and the association of rs10889677 met the Bonferroni correction cut-off for multiple testing (OR = 1.293, 95% CI = 1.034–1.61, *P* = 0.024, Table 4).

**Table 4:** Allele and genotype frequencies of *IL-23R* in CP with GDD and the control group.

**Group**	**Allele frequency**	***P* value**	**OR (95% CI)**	**Genotype frequency**	***P* value**	**Group**	**Allele frequency**	***P* value**	**OR (95% CI)**
rs10889657	C	T			C/C	C/T	T/T		
CP	132 (0.253)	390 (0.747)	0.06	0.801 (0.635–1.009)	23 (0.088)	86 (0.330)	152 (0.582)	0.026^a^	0.038
Control	372 (0.297)	880 (0.703)			53 (0.085)	266 (0.425)	307 (0.490)		0.665
rs6682925	C	T			C/C	C/T	T/T		
CP	239 (0.424)	325 (0.576)	0.578	0.946 (0.777–1.151)	54 (0.191)	131 (0.465)	97 (0.344)	0.608	0.412
Control	634 (0.437)	827 (0.563)			139 (0.189)	365 (0.497)	231 (0.314)		0.807
rs184444	G	T			G/G	G/T	T/T		
CP	196 (0.343)	376 (0.657)	0.279	0.894 (0.731–1.094)	38 (0.133)	120 (0.420)	128 (0.448)	0.201	0.246
Control	556 (0.368)	954 (0.632)			97 (0.128)	362 (0.479)	296 (0.392)	0.402	
rs1004819	A	G			A/A	A/G	G/G		
CP	219 (0.592)	151 (0.408)	0.021^b^	1.328 (1.044–1.688)	71 (0.384)	77 (0.416)	37 (0.200)	0.051	0.06
Control	544 (0.522)	498 (0.478)			150 (0.288)	244 (0.468)	127 (0.244)		0.16
rs11805303	C	T			C/C	C/T	T/T		
CP	232 (0.403)	344 (0.597)	0.019^c^	0.793 (0.652–0.963)	53 (0.184)	126 (0.438)	109 (0.378)	0.051	0.124
Control	696 (0.460)	818 (0.540)			167 (0.221)	362 (0.478)	228 (0.301)	0.304	
rs10889677	A	C			A/A	A/C	C/C		
CP	439 (0.767)	133 (0.233)	0.024^d^	1.293 (1.034–1.619)	174 (0.608)	91 (0.318)	21 (0.073)	0.004^e^	0.067
Control	1,079 (0.718)	423 (0.282)			378 (0.503)	323 (0.430)	50 (0.067)		0.084

P value after Bonferroni

a *0.077*,

b *0.126*,

c *0.114*,

d *0.144*,

e *0.024*.

Furthermore, we analyzed the associations between *IL-23R* and CP subtypes with various risk factors. There were significant differences in the rs11805303 allele frequency and the rs10889677 genotype frequency between controls and CP patients with NE; the association of rs10889677 with CP + NE remained significant even after Bonferroni correction (*OR* = 1.176, 95% CI = 0.947–1.460, *P* = 0.024, Table 5). There were no significant differences in either allele or genotype frequencies of the other SNPs in the other CP subgroups defined by clinical phenotypes and available risk factors (Supplementary Table 1).

**Table 5:** Allele and genotype frequencies of IL-23R in CP with NE and the control group.

**Group**	**Allele frequency**	***P* value**	**OR (95% CI)**	**Genotype frequency**	***P* value**	**Group**	**Allele frequency**	***P* value**	**OR (95% CI)**
rs10889657	C	T			C/C	C/T	T/T		
CP	146 (0.271)	392 (0.729)	0.271	0.881 (0.703–1.104)	25 (0.093)	96 (0.357)	148 (0.550)	0.163	0.109
Control	372 (0.297)	880 (0.703)			53 (0.085)	266 (0.425)	307 (0.490)		0.665
rs6682925	C	T			C/C	C/T	T/T		
CP	257 (0.425)	347 (0.575)	0.619	0.952 (0.787–1.154)	48 (0.159)	161 (0.533)	93 (0.308)	0.434	0.116
Control	634 (0.437)	827 (0.563)			139 (0.189)	365 (0.497)	231 (0.314)		0.807
rs184444	G	T			G/G	G/T	T/T		
CP	219 (0.369)	375 (0.631)	0.984	1.002 (0.823–1.220)	36 (0.121)	140 (0.495)	114 (0.384)	0.891	0.276
Control	556 (0.368)	954 (0.632)			97 (0.128)	362 (0.479)	296 (0.392)		0.402
rs1004819	A	G			A/A	A/G	G/G		
CP	201 (0.552)	163 (0.448)	0.322	1.128 (0.888–1.434)	62 (0.341)	77 (0.423)	43 (0.236)	0.391	0.051
Control	544 (0.522)	498 (0.478)			150 (0.288)	244 (0.468)	127 (0.244)		0.16
rs11805303	C	T			C/C	C/T	T/T		
CP	244 (0.405)	358 (0.595)	0.023^a^	0.801 (0.661–0.970)	52 (0.173)	140 (0.465)	109 (0.362)	0.083	0.541
Control	696 (0.460)	818 (0.540)			167 (0.221)	362 (0.478)	228 (0.301)		0.304
rs10889677	A	C			A/A	A/C	C/C		
CP	450 (0.750)	150 (0.250)	0.141	1.176 (0.947–1.460)	177 (0.590)	96 (0.320)	27 (0.090)	0.004^b^	0.011
Control	1,079 (0.718)	423 (0.282)			378 (0.503)	323 (0.430)	50 (0.067)		0.084

P value after Bonferroni

a *0.138*,

b *0.024*.

## Discussion

CP is a heterogeneous condition with multiple causes (Badawi and Keogh, 2013). The etiology in an individual patient is often multifactorial. These known CP causes, such as periventricular leukomalacia (PVL), NE, infarct, and premature delivery, account for only a minority of the total cases (Cowan et al., 2003; Yildiz et al., 2012; Colver et al., 2014; Chang et al., 2015). A single severe adverse event can be sufficient to cause CP, but much more often it is not a single cause, rather multiple concurrent risk factors that precede CP (Hankins and Speer, 2003; Djukic et al., 2009). Secondary neuroinflammation is associated with many CP risk factors. Findings from animal and clinical studies suggested that persistent neuroinflammation might prevent regeneration or exacerbate brain damage (Elovitz et al., 2011). Altered inflammation is one of the common causes of CP (Chiricozzi et al., 2011; Chen et al., 2013; Xia et al., 2018). Although the exact cause of CP is largely unknown, it is thought to be due to a combination of an altered fetal inflammatory response and primary brain damages.

Abnormal inflammation is one of the important pathological causes of immature brain injury (Djukic et al., 2009; Du et al., 2011), which is involved in the pathogenesis of central nervous system diseases, such as epilepsy, Parkinson's disease, cerebral ischemia, and hemorrhage, and may easily lead to secondary brain insult. During cerebral ischemia–reperfusion injury, inflammatory cells, such as microglia, astrocytes, and leukocytes, are activated. The activated inflammatory cells synthesize and secrete inflammatory mediators, phenomena that, in turn, further activate inflammatory cells and aggravate brain injury (Moon et al., 2009; Albertsson et al., 2018).

Some inflammatory cytokines are involved in immune responses, which cause brain injury through an inflammatory mechanism (McAdams and Juul, 2012). Several inflammatory cytokines have been identified as being involved in CP or other neurodevelopmental disorders, such as *IL-6, IL-8*, and *IL-17* (Chiricozzi et al., 2011; Chen et al., 2013). Our previous studies found that rs1800795 (G174C), located in the *IL-6* promoter region, was significantly associated with CP, and that the risk of spastic hemiplegia and quadriplegia in carriers of the rs1800795 C allele also significantly increased (Djukic et al., 2009; Wu et al., 2009). Central nervous system inflammation is often characterized by microglia activation, and active microglia will mediate neurotoxicity by secreting inflammatory cytokines, proteins, or other bioactive substances, resulting in secondary brain injury (Tang and Le, 2016).

**Figure 3:**
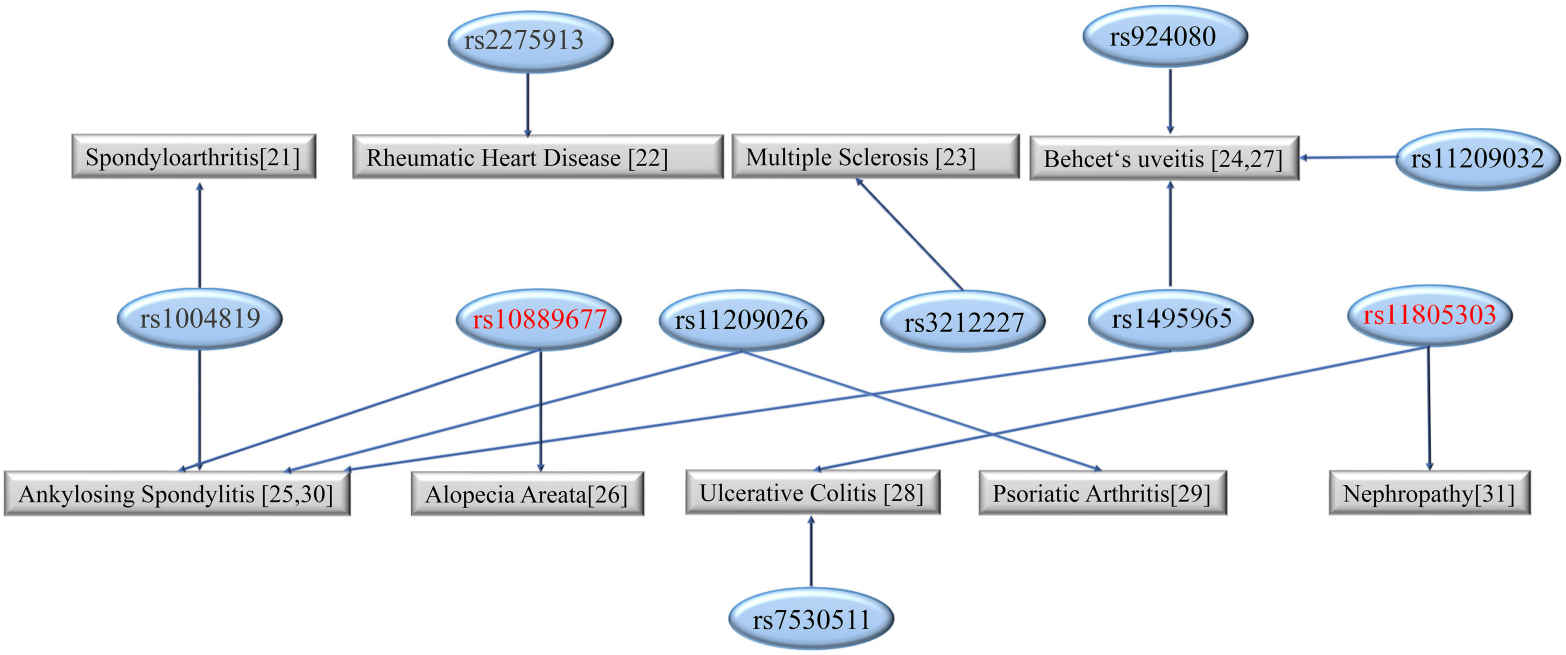
The diseases linked to different sites of *IL-23R*. The SNPs studied in the present study are marked in red.

A recent study demonstrated that inflammatory factors are related to microglia activation (Zhao et al., 2020). IL-23 mainly acts as a pro-inflammatory cytokine and has potential anti-tumor and anti-infection effects. IL-23R can activate Janus kinase (JAK). IL-23 bound IL-23R can active downstream JAKs and phosphorylate the signal transducer and activator of transcription (STAT) binding site in the intracellular region of the receptor. STAT dimerizes and is phosphorylated by JAKs. The phosphorylated STAT dimers enter the nucleus and act on downstream target genes. *IL-23R* has been identified to associate with multiple diseases, including alopecia areata (rs10889677) and nephropathy (rs10805303) (Figure 3) (Safrany et al., 2011; Yu et al., 2012; Huang et al., 2016; Qin et al., 2016; Poomarimuthu et al., 2018; Sode et al., 2018; Zhong et al., 2018; Kramer et al., 2019; Loures et al., 2019; Ruyssen-Witrand et al., 2019; Tabatabaei-Panah et al., 2020).

Our results showed that *IL-23R* rs10889677 increases susceptibility to CP at the overall level and in some subgroups. These findings suggest that *IL-23R* is a potential susceptibility gene for CP. Furthermore, studies have shown that rs10889677 is related with different diseases in different races, such as Crohn's disease in both Jewish and non-Jewish populations, ankylosing spondylitis in Caucasian patients, Graves' disease in North Americans, and ulcerative colitis in Europeans (Duerr et al., 2006; Wellcome Trust Case Control et al., 2007; Brown, 2008; Huber et al., 2008; Silverberg et al., 2009). Therefore, we have sufficient reason to assume the positive association of *IL-23R* gene with the CP etiology. Whether *IL-23R*, as a gene associated with inflammatory bowel disease, can also affect the brain development of children by affecting intestinal flora remains to be seen.

Given that *TNF-*α and *IL-6* are significantly increased after brain injury (Leviton and Dammann, 2004; Xie et al., 2014), we hypothesize that IL-23R, like other inflammatory factors *TNF-*α and *IL-6*, may cause brain damage and lead to CP through the following steps. (1) Increased inflammatory cytokines promote the release of nitric oxide synthase and free radicals and excitatory amino acids, which have toxic effects on neurons, especially developing brain tissue. (2) They will trigger a systemic inflammatory response that leads to brain damage when undergoing intrauterine infection. (3) Endothelial cell damage can cause thrombosis. Inflammatory factors will activate platelets, lead to their aggregation, activate coagulation factors, and damage white matter neurons. (4) The damage will increase the permeability of the blood–brain barrier, thereby allowing peripheral bacteria and inflammatory factors to enter the brain, aggravating brain damage. (5) They promote the release of prostaglandins and other substances, resulting in pregnancy's advance labor, leading to an increased risk of CP.

Our study has some limitations. First, this is a study based on a single gene for susceptibility to CP. Given the genetic heterogeneity and gene–gene interaction involved in the CP etiology, other candidate genes that are part of the IL-23R signaling pathway need to be analyzed together. Second, we were unable to measure IL-23R protein expression in the brains of the subjects in the current study; future studies are encouraged to examine the inflammatory cytokine alteration in the brain. Third, although our study demonstrated an association between the *IL-23R* rs10889677 SNP and CP, further functional and replicated studies are necessary to verify the association of *IL-23R* with CP, which is of great significance to identify the CP etiology and pathogenesis.

In summary, a significant association between *IL-23R* and CP was firstly detected in Han Chinese, suggesting that the *IL-23R* gene has a significant effect on the risk of CP, especially in subjects with GDD or NE. The inflammatory response and cytokine cascade are likely to play a role in the occurrence and development of CP. This result needs to be further validated with well-designed studies with large sample sizes and in other populations. We should also pay attention to the possibility of increased risk of CP if the fetus is found to carry the *IL-23R* risk genotypes before or after delivery.

## Data Availability Statement

The raw data supporting the conclusions of this article will be made available by the authors, without undue reservation.

## Ethics Statement

The studies involving human participants were reviewed and approved by the Ethics Committee of Zhengzhou University. Written informed consent to participate in this study was provided by the participants' legal guardian/next of kin.

## Author Contributions

QX and CZ conceived and designed the study. YX, JS, LX, QS, CG, and DZ recruited subjects and sorted out clinical information. YW and DB performed all of the laboratory work. YW and YF performed all data and statistical analyses. YF drafted the manuscript, and QX, CZ, XW, YW, and YX revised the manuscript critically for important intellectual content. YQ and JS provided data, developed models, reviewed results, and provided guidance on the methods. All authors contributed and critically reviewed the final version of the manuscript. All authors have read and approved the final manuscript.

## Conflict of Interest

The authors declare that the research was conducted in the absence of any commercial or financial relationships that could be construed as a potential conflict of interest. The reviewer HB-P declared a past co-authorship with the authors XW and CZ to the handling editor.
